# Non-Isothermal Melt Crystallization of a Biodegradable Polymer Studied by Two-Dimensional Infrared Correlation Spectroscopy

**DOI:** 10.3390/molecules30051131

**Published:** 2025-03-01

**Authors:** Isao Noda

**Affiliations:** Department of Materials Science and Engineering, University of Delaware, Newark, DE 19716, USA; noda@udel.edu; Tel.: +1-513-600-9255

**Keywords:** poly(hydroxyalkanoate), two-dimensional correlation infrared spectroscopy, melt crystallization

## Abstract

The non-isothermal melt crystallization process of poly[(R)-3-hydroxybutyrate-*co*-(R)-3-hydroxyhexanoateate] (PHBHx) was monitored using attenuated total reflection infrared (ATR IR) measurement. The resulting time- and temperature-dependent spectra were subjected to the two-dimensional correlation spectroscopy (2D-COS) analysis. The C=O stretching region of the PHBHx sample consisted of several distinct IR contributions attributable to the population of amorphous component, well-ordered type I lamellar crystal, and less ordered inter-lamellar type II crystal. The spectral intensity change in type I crystal occurs in the earlier stage of the crystallization at a higher temperature range compared to the overall intensity decrease in the amorphous component occurring throughout the crystallization process. The growth of the type II crystal started in a later stage at a lower temperature than the creation of the type I crystal. An early decrease in a small but distinct portion of the amorphous component may be related to a crystallization precursor species with some level of molecular order. Hetero-mode correlation analyses revealed that the crystalline band intensity changes in the C-H stretching and fingerprint regions all occur later than the population changes in crystalline species reflected by the carbonyl stretching bands. This observation suggests that the spectral intensity changes in the C-H stretching and fingerprint regions do not directly represent the population dynamics of the crystalline and amorphous species but probe instead the molecular state of the crystalline entities still undergoing the evolutionary changes.

## 1. Introduction

Professor Jaan Laane has been a true intellectual leader conducting the research effort in the field of molecular spectroscopy, including Fourier transform infrared (FT-IR), fluorescence, and laser Raman spectroscopy. His work has greatly contributed to the understanding of molecular vibrations and structures. He has published a number of articles and edited books on the subject of molecular spectroscopy. This author had the privilege and pleasure of contributing chapters to his books [1,2], which included some discussions on the IR study of a bio-based biodegradable polymer. The current article is a continuation of the molecular spectroscopy work on a similar bioplastics system to further explore the in-depth analysis of the material.

Polyhydroxyalkanoates or PHAs are totally bio-based and biodegradable aliphatic semicrystalline polyesters that have been explored as possible alternative materials to replace conventional petroleum-based plastics [3,4,5,6,7]. Among many types of PHAs, the interest in poly[(R)-3-hydroxybutyrate-*co*-(R)-3-hydroxyhexanoateate] or PHBHx has especially increased. Because of the molecular architecture of this copolymer, PHBHx possesses a unique set of practically useful properties, like ductility, toughness, and superior processing compatibility [8,9]. The material is nowadays produced by a large scale industrial manufacturing process using the fermentation of renewable resources, like vegetable oils. Because PHBHx has a set of attractive physical properties similar to those of conventional plastics made from petroleum, the additional unique property of biodegradability has attracted keen interest as a potentially useful sustainable alternative material. Melt processing is the preferred processing method for thermoplastics, like PHBHx, where the material is heated to become soft and fluid and then cooled to solidify in the shape of the final products. For a semicrystalline polyester, understanding the detailed crystallization process becomes a critical requirement for controlling the effective solidification and subsequent properties of the products.

As Laane has repeatedly demonstrated in his professional life, spectroscopy is an excellent tool to track the important but sometimes complex dynamics of various physical and chemical processes, including polymer crystallization, at the molecular level in order to provide useful insights into mechanistic understanding. Among many commonly available spectroscopic techniques, attenuated total reflectance (ATR) infrared (IR) spectroscopy is well suited for the in situ monitoring of thermal transition processes, like the crystallization of a polymer from the melt. While the crystallization of a PHAs melt has often been studied under the idealized isothermal condition [10,11], a more realistic melt crystallization process for industrial polymers is typically carried out under non-isothermal conditions with both varying temperatures and time. Although such time- and temperature-dependent ATR IR measurements can provide high-quality spectroscopic data that are rich in the latent information content, analysing and interpretating the very complex and highly convoluted results often become a major challenge.

Two-dimensional correlation spectroscopy (2D-COS) is a versatile data analysis tool employed by many researchers [12,13,14]. Evolution patterns of spectral intensities induced by the application of external perturbation, such as thermal processing, are systematically analyzed using a form of cross correlation approach. It was noted that certain parts of a spectrum can change simultaneously in a coordinated fashion, while others may vary differently in out-of-phase sequences. Such characteristic variation patterns are conveniently displayed in the form of planar spectral mapping schemes defined by two independent wavenumber axes. By spreading the overlapped bands along the second dimension, apparent spectral resolution is often enhanced. By following a simple set of interpretation rules for correlation peaks appearing in the 2D-COS maps, one can deduce the streamlined mechanistic picture of the evolving system represented by the spectra. In this work we apply the 2D-COS analysis to the ATR IR spectra of a bioplastic PHBHx sample undergoing a non-isothermal melt crystallization process to elucidate the intricate formation steps of crystalline components, which eventually control the final performance properties of the products.

## 2. Background

A very brief overview is provided here for the 2D correlation spectroscopy (2D-COS) to be applied toward the time- and temperature-dependent ATR IR spectra obtained during the non-isothermal melt crystallization of a PHBHx sample. A more detailed description of this general technique is available elsewhere [1,2,12,13,14]. Consider a set of *m* IR spectra $A(\tilde{\nu },t_{i})$, with i=1, 2,⋯m, which are measured as functions of wavenumber $\tilde{\nu }$ and another well-defined physical variable $t_{i}$, such as time or temperature; we define the dynamic spectrum $\tilde{A}(\tilde{\nu },t_{i})$ as

(1) $$\tilde{A}\left(\tilde{\nu },t_{i}\right)=A\left(\tilde{\nu },t_{i}\right)-\bar{A}(\tilde{\nu })$$

The reference spectrum $\bar{A}(\nu )$ is typically set to be the average spectrum taken during the observation interval between $t_{1}$ and $t_{m}$.

(2) $$\bar{A}\left(\tilde{\nu }\right)=\frac{1}{m}\sum_{i=1}^{m}A\left(\tilde{\nu },t_{i}\right)$$

The synchronous and asynchronous 2D correlation spectra, $\Phi ({\tilde{\nu }}_{1},\;{\tilde{\nu }}_{2})$ and $\Psi ({\tilde{\nu }}_{1},\;{\tilde{\nu }}_{2})$, for $\tilde{A}\left(\tilde{\nu },t_{i}\right)$ are given by

(3) $$\Phi \left({\tilde{\nu }}_{1},{\tilde{\nu }}_{2}\right)=\frac{1}{m-1}\sum_{i=1}^{m}\tilde{A}\left({\tilde{\nu }}_{1},t_{i}\right)\cdot \tilde{A}\left({\tilde{\nu }}_{2},t_{i}\right)$$

(4) $$\Psi \left({\tilde{\nu }}_{1},{\tilde{\nu }}_{2}\right)=\frac{1}{m-1}\sum_{j=1}^{m}\tilde{A}\left({\tilde{\nu }}_{1},t_{j}\right)\cdot \sum_{i=1}^{m}N_{ij}\tilde{A}\left({\tilde{\nu }}_{2},t_{i}\right)$$

with the elements of the Hilbert–Noda transformation matrix defined as

(5) $$N_{ij}=\left\{\begin{array}{cc} \frac{1}{\pi (j-i)} & if\;i\neq j\\ 0 & otherwise \end{array}\right.$$

Synchronous spectrum $\Phi \left({\tilde{\nu }}_{1},\;{\tilde{\nu }}_{2}\right)$ corresponds to the covariance of spectral intensity variations. It characterizes the simultaneous or coordinated changes in band intensities at two IR wavenumbers ${\tilde{\nu }}_{1}$ and ${\tilde{\nu }}_{2}$. The magnitude of the so-called autopeak appearing at the main diagonal position, i.e., ${\tilde{\nu }}_{1}={\tilde{\nu }}_{2}$, represents the extent or variance of the spectral intensity at a given coordinate. Peaks appearing in the off-diagonal positions of a 2D-COS map are called cross peaks. If the sign of a synchronous cross peak at a coordinate position ${(\tilde{\nu }}_{1,}{\tilde{\nu }}_{2})\;$ is positive, i.e., $\Phi \left({\tilde{\nu }}_{1},\;{\tilde{\nu }}_{2}\right)>0$, the measured IR spectral intensities at ${\tilde{\nu }}_{1}$ and ${\tilde{\nu }}_{2}$ are either increasing or decreasing together in the same direction. The intensities are changing in opposite directions for a negative cross peak.

An asynchronous spectrum $\Psi \left({\tilde{\nu }}_{1},\;{\tilde{\nu }}_{2}\right)$, in turn, characterizes the out-of-phase or sequential changes in spectral intensities, which are presumably arising from different sources. Signals arising from the same source cannot vary independent of each other. Thus, the appearance of asynchronous cross peaks implies the presence of multiple species which are responding differently to the given perturbation, like temperature change. This feature also becomes useful in differentiating highly overlapped spectral bands if they are responding differently to the perturbation. Enhanced spectral resolution based on the asynchronous correlation is one of the unique advantages of 2D-COS analysis.

If the signs of both synchronous and asynchronous cross peaks at a given coordinate $\left({\tilde{\nu }}_{1},\;{\tilde{\nu }}_{2}\right)$ are the same, i.e., $\Phi \left({\tilde{\nu }}_{1},\;{\tilde{\nu }}_{2}\right)\cdot \Psi \left({\tilde{\nu }}_{1},\;{\tilde{\nu }}_{2}\right)>0$, spectral intensity changes measured at ${\tilde{\nu }}_{1}$ predominantly occur earlier than that at ${\tilde{\nu }}_{2}$ along the variable *t*. On the other hand, if the two peak signs are different, this relationship is reversed. Thus, the 2D-COS cross peaks immediately provide useful knowledge about the sequential order of spectral intensity changes. Such information obviously is beneficial in establishing a streamlined mechanistic insight into convoluted multistep processes.

## 3. Results and Discussion

### 3.1. ATR IR Spectra

**Figure 1:**
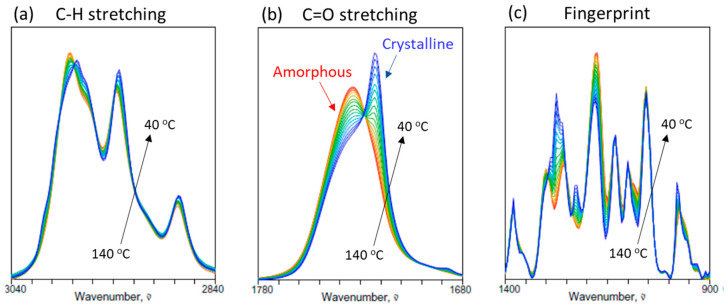
Temperature-dependent ATR IR spectra of PHBHx (12 mol% 3HHx) undergoing the non-isothermal melt crystallization process between 140 and 40 °C: (**a**) C-H stretching region, (**b**) C=O stret5ching region, and (**c**) fingerprint region.

Figure 1 shows the overlaid spectral plots of three pertinent regions of the ATR IR spectra of PHBHx (12 mol% 3HHx) undergoing the non-isothermal crystallization process from the amorphous melt at 140 °C to the semicrystalline state at 40 °C. The effect of the crystal growth at the expense of the amorphous melt component is well reflected by the changes in the spectral intensities of IR bands. For example, spectral variations in the C-H stretching region (Figure 1a) are dominated by the increasing contributions of IR intensities associated with the crystalline component around 2975, 2968, 2932 and 2875 cm^−1^ and decreasing contributions from the amorphous component around 2984 and 2942 cm^−1^. Moieties contributing to the molecular vibrations for these bands are obviously placed in different local environments characteristic to ordered crystal lattice and random amorphous coils.

The spectral region corresponding to the carbonyl stretching vibrations of the polyester (Figure 1b) provides the information most directly useful for tracking the crystallization of PHBHx. The completely molten PHBHx sample exhibits a single IR peak centered around 1736 cm^−1^ corresponding to the fully amorphous state of the polymer. Upon cooling the sample, an additional spectral feature appears at around 1720 cm^−1^, while the peak intensity at around 1736 cm^−1^ gradually decreases. The temperature of the appearance of the 1720 cm^−1^-band coincides with the onset of crystallization for this sample. It is, therefore, reasonable to assign the increasing feature around 1720 cm^−1^ to be associated with the contribution from the growing crystalline component population of the polymer. There remain enough amorphous features in the sample at 40 °C to indicate that this polymeric material is in the semicrystalline state at the final stage. It should be pointed out that the lower-wavenumber feature at around 1720 cm^−1^ actually originates from the enhanced hydrogen bonding interaction of carbonyl oxygen instead of the specific helical structure of polymer segments in the crystal lattice [15].

The fingerprint region of the IR spectrum (Figure 1c), primarily reflecting the contributions from C-C-O and C-O-C vibrations of PHBHx, also exhibits complex and rich information associated with the crystallization process. These bands are sensitive to the conformational state of the polymer backbone and the interactions of the neighboring segments. Similarly to the C=O stretching region, peaks associated with the growth of crystalline component increase their intensities at around 1389, 1276, 1263, and 1228 cm^−1^, while those for the amorphous component at around 1302, 1259, and 1181 cm^−1^ decrease. Unlike the carbonyl stretching region, however, these bands in the fingerprint region coexist in a highly congested and overlapped manner to make the description of crystallization population dynamics much less straightforward.

### 3.2. 2D-COS Analysis of the C=O Stretching Region

**Figure 2:**
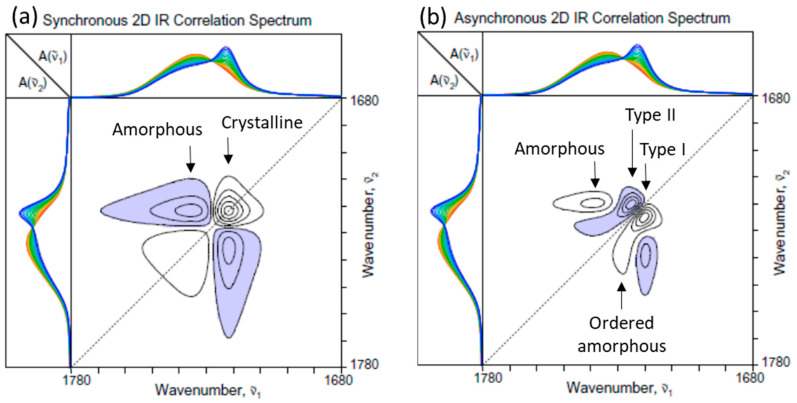
(**a**) Synchronous and (**b**) asynchronous 2D-COS spectra of PHBHx undergoing the melt crystallization process constructed from the set of 1D ATR IR spectra in the C=O stretching region (Figure 1b). One-dimensional spectra are provided at the top and left side of each 2D map, and negative cross peaks are indicated by the blue shading.

Figure 2 shows the 2D-COS spectra constructed from the set of time- and temperature-dependent ATR IR spectra of PHBHx undergoing melt crystallization in the C=O stretching region (Figure 1b). The overlaid 1D spectra are provided at the top and left side of each 2D map for reference purposes, and negative cross peaks are indicated by the blue shading. A similar system was actually analyzed using 2D-COS in the past [1,2] but without the intensity correction by normalization, which has brought out the details of the crystalline and amorphous contributions more clearly this time. The synchronous spectrum (Figure 2a) shows the autopeaks along the main diagonal positions at around 1736 and 1722 cm^−1^, which, respectively, match well with the major band positions of the amorphous and crystalline contributions. In parallel, there appear a pair of cross peaks at the corresponding spectral coordinates. The negative signs of the cross peaks indicate that the spectral intensity of the band around 1736 cm^−1^ is decreasing, while the other band around 1722 cm^−1^ is increasing. This observation agrees with the fact that the population of the amorphous component of PHBHx is decreasing while the crystalline contribution is increasing during the melt crystallization process.

The asynchronous 2D-COS spectrum (Figure 2b) provides another interesting story. First of all, the spectrum itself exhibits a number of asynchronous cross peaks, indicating that the spectral intensity changes in this system during the melt crystallization are not simultaneously proceeding in a simple coordinated manner. The presence of asynchronous cross peaks around the coordinates of (1736, 1720) and (1720, 1736) indicates that the decrease in the amorphous component reflected by the declining behavior of the 1736 cm^−1^ band does not occur simultaneously with the increase in the major crystalline contribution from the band around 1720 cm^−1^. A comparison of the peak signs between the synchronous and asynchronous cross peaks reveals that the intensity increase around 1720 cm^−1^ occurs predominantly in earlier stages at a higher temperature while the decrease around 1736 cm^−1^ continues to the later stage of the lower temperature range.

The reason for the asynchronicity between the crystalline 1720 cm^−1^ band and the amorphous 1736 cm^−1^ band may be understood by the appearance of an additional cross peak pair around (1720, 1724) and (1724, 1720). It appears that there are two distinct populations of crystalline contributions, such that the amorphous component is further consumed to create the second crystalline component at 1724 cm^−1^ even after the growth of the 1720 cm^−1^ component has slowed down. The presence of two types (I and II) of crystalline components during the melt crystallization of PHBHx has been observed in the past [1,2,15,16,17,18,19]. The type I crystal found at around 1720 cm^−1^, which grows first, is believed to have a well-ordered lattice structure, corresponding most likely to the volume-filling lamellae of the crystalline spherulites. In turn, the type II crystal, which grows later, is speculated to have a less ordered structure associated with the secondary crystals formed within the confined inter-lamellar spacing. While the two types of PHBHx crystals exhibiting distinct growth behaviors are vividly revealed in the asynchronous 2D-COS plot (Figure 2b), it is actually rather difficult to detect the difference by a cursory observation of the set of 1D spectra, like the one shown in Figure 1b.

The asynchronous cross peak at (1720, 1724), exhibiting the foothill-like extension toward the region at around (1730, 1736), presents an interesting observation. This feature becomes visible only if the threshold of the contour line level is set sufficiently low, such that the existence of the additional peak was not always noticed in the past [1,2]. A previous Raman study of the isothermal crystallization of a similar PHBHx sample also observed a similar feature at around 1734 cm^−1^ [20]. While this band was tentatively assigned to the nucleating crystals with imperfect structures, this assignment may not be applicable here. The IR intensity at 1730 cm^−1^ definitely decreases during the crystallization, so it is reasonable to assign this band to be a part of the amorphous contribution. However, the decline of this band intensity occurs earlier than that of the rest of the amorphous region, and its behavior is actually well synchronized with the increase in the intensity of the type I crystal at around 1720 cm^−1^. It is tempting to speculate that this band at 1730 cm^−1^ is related to a sort of precursor species for the formation of the well-ordered lamellar type I crystal. Because of the lower-wavenumber position of this band compared to the rest of the amorphous band, it is possible that a somewhat more ordered non-crystalline species may be present in the melt before the crystal formation.

### 3.3. 2D-COS in Other Spectral Regions

**Figure 3:**
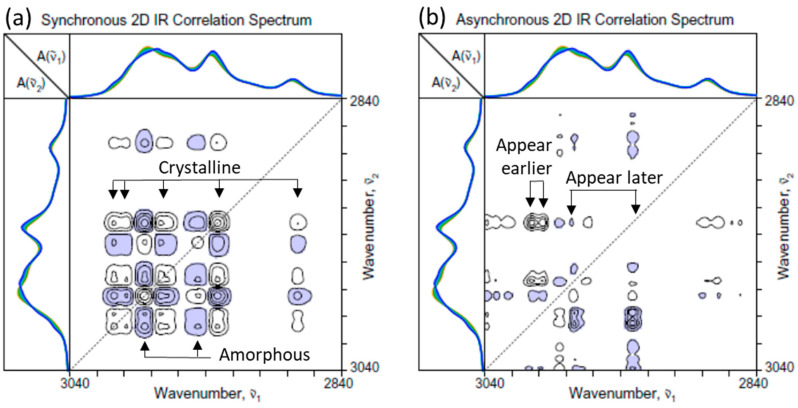
(**a**) Synchronous and (**b**) asynchronous 2D-COS spectra of PHBHx undergoing the melt crystallization process constructed from the set of ATR IR spectra in the C-H stretching region (Figure 1a).

Figure 3 shows the 2D-COS spectra constructed from the ATR IR spectra in the C-H stretching region (Figure 1a). The spectral intensity changes in this region show the time- and temperature-dependent dynamics of the increasing crystalline and decreasing amorphous contributions. Major increasing bands, connected by the positive synchronous cross peaks, are assignable to the symmetric methyl stretching mode at around 2875 cm^−1^ and antisymmetric methylene stretching at around 2932 cm^−1^. They are clearly associated with the crystalline component. In turn, the decreasing band around 2942 cm^−1^ must be arising from the contribution of the amorphous component. An asymmetric methyl stretching contribution of crystal is observed at around 2975 cm^−1^, as well as the amorphous band at around 2984 cm^−1^. In addition, a pair of crystalline bands at around 2996 and 3007 cm^−1^ are observed. They are believed to arise from an unusual localized interaction between one of the methyl hydrogens and carbonyl oxygen, as observed in [21,22,23,24]. While C–H hydrogens do not usually participate in the hydrogen bonding interactions, the close proximity of the methyl hydrogen and carbonyl oxygen in the crystalline lattice seems to bring in this unique interaction.

The fact that the asynchronous spectrum (Figure 3b) exhibits a number of cross peaks indicates that the spectral intensity variations associated with various components in this region once again do not all occur uniformly. For example, the intensities of the two high-wavenumber crystalline bands at 2996 and 3007 cm^−1^ seem to increase well ahead of all the other bands. The increases in the rest of the bands associated with the crystalline contributions occurring later at a lower temperature and decreases in the bands at around 2984 and 2942 cm^−1^ for the amorphous component. The increases in the intensities at around 2975 and 2932 cm^−1^ occur later than that of the band at around 2968 cm^−1^. Unlike the carbonyl stretching region, however, it is rather difficult to assign highly overlapped bands with increasing intensities in this region to any specific crystalline species.

**Figure 4:**
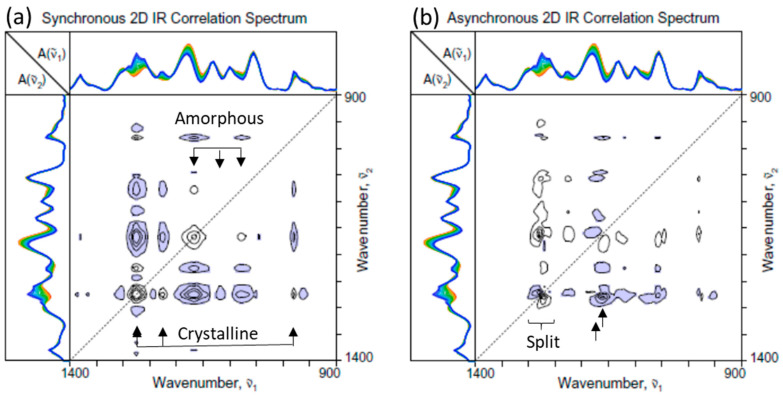
(**a**) Synchronous and (**b**) asynchronous 2D-COS spectra of PHBHx undergoing the melt crystallization process constructed from the set of ATR IR spectra in the fingerprint region (Figure 1c).

Figure 4 shows the 2D-COS spectra constructed from the spectra in the fingerprint region (Figure 1c). Once again, bands associated with both increasing and decreasing components of PHBHx during the crystallization are observed. Even though many band profiles tend to overlap, the signs of cross peaks in the synchronous spectrum (Figure 4a) makes it possible for the initial classification of the pertinent bands. The intensities of two bands at around 1170 and 1080 cm^−1^ arising from the ester C-O and C-O-C stretching vibrations are decreasing. They are presumably assignable to the amorphous component, while other increasing bands are assignable to the crystalline contributions. The most prominent band is the increasing C-O-C stretching band at around 1276 cm^−1^, which splits into two in the corresponding asynchronous spectrum (Figure 4b). The asynchronous spectrum also shows that the amorphous ester C-O stretching band at around 1170 cm^−1^ is accompanied by an overlapping adjacent crystalline band at around 1181 cm^−1^. The enhanced resolution of the overlapped bands, achieved by spreading the peaks along the second dimension, is one of the key advantages of 2D-COS analysis.

### 3.4. Hetero-Mode 2D-COS

While the resolution of overlapped bands arising from different contributing species obviously is a beneficial result by itself, proper classification and assignment of such individual spectral responses to specific contributing components of the system are even more desired. Hetero-mode correlation among different vibrational modes of the sample becomes a useful tool to achieve such a task. Hetero-mode correlation is the simplest form of the so-called 2D hetero-spectral correlation analyses. In hetero-spectral correlation, two sets of spectral datasets observed under the same perturbation conditions are compared. For example, IR and Raman spectra may be compared to each other to take advantage of the different sensitivities of individual probes to particular molecular responses. If one type of the dataset is better understood or better interpreted than the other, one can use the former to assist the analysis of the latter. Thus, hetero-spectral correlation is sometimes called the Rosetta Stone analysis, based on the historical discovery of a tablet written in previously less understood Egyptian hydrographs in parallel with the interpretable Greek text.

**Figure 5:**
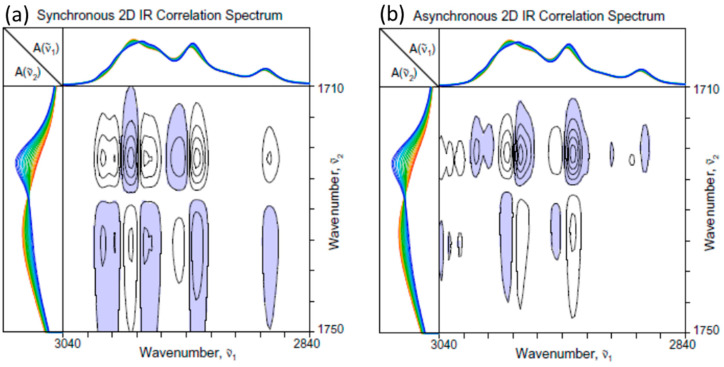
(**a**) Synchronous and (**b**) asynchronous 2D hetero-mode correlation spectra of PHBHx undergoing the melt crystallization process constructed by comparing the ATR IR spectra in the C-H stretching region (Figure 1a) and C=O stretching region (Figure 1b).

Figure 5 shows the hetero-mode correlation between the C=O stretching and C-H stretching regions of the ATR IR spectra. We have already established that the carbonyl stretching mode of this PHBHx sample consists of several distinct contributions, such as the amorphous band at around 1736 cm^−1^, well-ordered type I lamellar crystals at around 1720 cm^−1^, and less ordered type II inter-lamellar crystals at around 1724 cm^−1^. The hetero-mode synchronous spectrum (Figure 5a) clearly reveals the specific correlations among crystalline and amorphous bands in the two different spectral regions. The vertical ${\tilde{\nu }}_{2}$ coordinates of all the maxima and minima of the synchronous peaks are observed at around 1736 and 1723 cm^−1^. This result may seemingly suggest that predominant crystalline changes in the C-H stretching region are associated more so with the growth of less ordered type II crystals. The asynchronous spectrum (Figure 5b) also supports this tentative conclusion, since the peak maxima of the crystalline bands in the C-H stretching region are all found at around ${\tilde{\nu }}_{2}=$1720 cm^−1^, indicating that they are behaving somewhat differently from the formation of well-ordered type I crystals. On the other hand, the closer peak position of 1723 cm^−1^ instead of 1724 cm^−1^ also may suggest that signals are arising from both type I and type II crystals.

The sequential order analysis based on the cross peak signs of the hetero-mode synchronous and asynchronous spectra reveals a fascinating discovery. It was revealed that intensity changes in bands in the C-H stretching region all seem to happen later at a lower temperature range than those of the carbonyl stretching region. In other words, intensity variations in the C-H stretching region bands occur after the type I and II crystals are already formed. The band intensity changes, therefore, may not directly represent the formation of different species of crystals but rather reflect the subtle evolutionary rearrangement of the crystalline lattice structure. Gradual reorganization of the lamellar structure of PHBHx crystals toward higher perfection was indeed suggested in the past, based on a low-frequency Raman study of the isothermal crystallization process of PHBHx [20]. If this conjecture is correct, we may now have a potentially useful IR probe in the C-H stretching region for tracking the state of crystalline lattice structure evolution.

**Figure 6:**
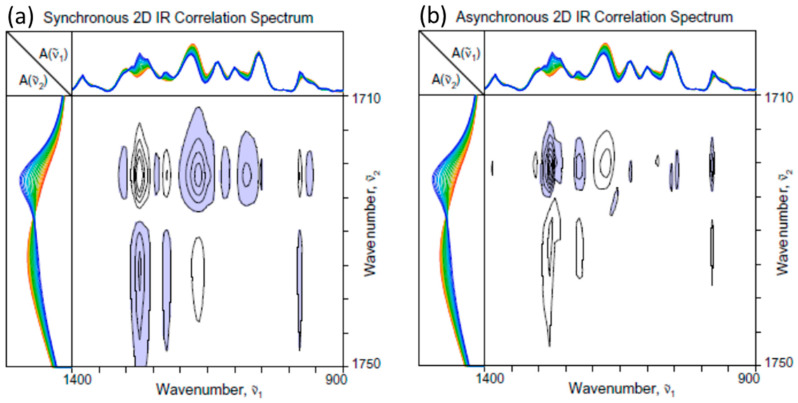
(**a**) Synchronous and (**b**) asynchronous 2D hetero-mode correlation spectra of PHBHx undergoing the melt crystallization process constructed by comparing the ATR IR spectra in the fingerprint region (Figure 1c) and C=O stretching region (Figure 1b).

Figure 6 shows the hetero-mode correlation between the fingerprint and C=O stretching region. Once again, the asynchronous spectrum (Figure 6b) suggests that the crystalline signals in the fingerprint region are behaving somewhat differently from the well-ordered type I crystal at around 1720 cm^−1^ of the C=O stretching region. Compared to the C-H stretching region, however, the ${\tilde{\nu }}_{2}$ coordinates of the synchronous cross peaks for the apparent crystalline contributions in the fingerprint region are even closer to 1722 cm^−1^. The result suggests that the IR signals increasing during the crystallization process in the fingerprint region are not necessarily originating from a distinct population of the crystalline species.

The sequential order analysis based on the cross peak signs once again reveals that the band intensity changes in the fingerprint region are all occurring later than those in the carbonyl stretching region in a manner similar to the C-H stretching bands. The variations in band intensities in the fingerprint region are observed after the type I and II crystals are already formed. The nature of the spectral changes in this region, however, may be somewhat different. While the C-H stretching mode vibrations are more likely influenced by the perfection of crystalline lattice packing, the fingerprint region probably has higher susceptibility toward the conformational order of polymer chains and inter-chain interactions.

## 4. Experimental

### 4.1. Material

The sample of poly([R]-3-hydroxybutyrate-*co*-[R]-3-hydroxyhexanoate) (PHBHx) was prepared in the research laboratory of the Procter & Gamble Company in Cincinnati, OH, USA, by the fermentation of palm kernel oil using a strain of Gram-negative bacterium *Aeromonas caviae*. The polymer was extracted from the cell body using chloroform as the solvent and purified by repeated precipitation and dissolution using ethanol and hexane as the precipitating non-solvent. The content of comonomer 3-hydroxyhexanoate in the comonomer was about 12 mol%, with the rest being 3-hydroxybutyrate. The weight average molecular weight was about 894,000.

### 4.2. Method

The PHBHx sample was heated to 170 °C, which is well above the melt temperature of this copolymer at about 120 °C, and kept for one minute. The molten polymer sample was placed in the temperature-controlled attenuated total reflection (ATR) cell, initially kept at the starting temperature of 140 °C, and mounted on a Bio-Rad Model 165 FT-IR spectrometer (Hercules, CA, USA). The ATR IR spectra of the PHBHx sample were continuously collected while the temperature of the ATR cell was decreased at a constant cooling rate of 3.2 °C/min from 140 °C to 40 °C. The whole process took less than 30 min to complete. The collected spectra were normalized by the area under the curve between 1780 and 1760 cm^−1^ to minimize the superfluous interferences, such as sample contact effect. The correction factor for the normalization was only within 5% of the original data, but the treatment makes it easier to reveal the detailed dynamics of the spectral contribution of the crystalline component.

## 5. Conclusions

The non-isothermal crystallization of PHBHx (12% 3HHx) by cooling the melt from 140 °C to 40 °C at the rate of 3.2 °C/min was monitored using ATR IR measurement. The resulting time- and temperature dependent spectra were subjected to a 2D-COS analysis. It was revealed that the C=O stretching region of the PHBHx sample consisted of several distinct IR contributions attributable to the population of an amorphous component detected at around 1736 cm^−1^, well-ordered type I lamellar crystal at around 1720 cm^−1^, and less ordered inter-lamellar type II crystal at around 1724 cm^−1^. The spectral intensity change in the type I crystal occurred in the earlier stage of the crystallization at a higher temperature range compared to the overall intensity change in the amorphous component occurring throughout the crystallization process. The growth of the type II crystal started at a stage much later than the creation of the type I crystal. In addition to the above observations, some early decreasing in a portion of the amorphous component was also observed at around 1730 cm^−1^. This amorphous component may be a precursor species to the crystallization with some level of molecular order.

Dynamics of the spectral intensity variations in the C-H stretching and fingerprint regions are much more complex, even though it is possible to classify bands to amorphous and crystalline contributions according to the directions of the intensity changes. Crystalline bands did not increase in their intensity in a uniform manner, and there are noticeable asynchronous behaviors of individual bands during the crystallization process. A hetero-mode correlation analysis of the band intensity changes in the C-H stretching and fingerprint regions with respect to the C=O stretching region revealed that they all occur later than the population changes in crystalline and amorphous components reflected by the carbonyl stretching bands. In other words, the spectral intensity changes in these regions are observed after the type I and II crystals are already formed. This discovery suggests that the spectral intensity changes in the C-H stretching and fingerprint regions do not directly represent the population dynamics of specific crystalline and amorphous species during the crystallization but probe instead the molecular state of the already created crystals still undergoing the evolutionary changes.

## Data Availability

Data are contained within the article.

## References

[1] Noda I., Laane J. (2009). Generalized two-dimensional correlation spectroscopy. Frontiers of Molecular Spectroscopy.

[2] Noda I., Laane J. (2018). Advances in two-dimensional correlation spectroscopy (2DCOS). Frontiers and Advances in Molecular Spectroscopy.

[3] Palmerio-Sámchez T., O’Flaherty V., Lens P.N.L. (2022). Polyhydroxyalkanoate bio-production and its rise as biomaterial of the future. J. Biotechnol..

[4] Koller M. (2022). Advances in polyhydroxyalkanoate (PHA) production, Volume 3. Bioengineering.

[5] Dalton B., Bhagabati P., De Micco J., Padamati R.B., O’Connor K. (2022). A review on biological synthesis of the biodegradable polyhydroxyalkanoates and the development of multiple applications. Catalysts.

[6] Kumar M., Rathour R., Singh R., Sun Y., Pandey A., Gnansounou E., Lin K.-Y.A., Tsang C.W., Thakur I.S. (2020). Bacterial polyhydroxyalkanoates: Opportunities, challenges, and prospects. J. Clean. Prod..

[7] Samui A.B., Kanai T.R. (2019). Polyhydroxyalkanoates based copolymers. Int. J. Biol. Macromol..

[8] Noda I., Green P.R., Satkowski M.M., Schechtman L.A. (2005). Preparation and properties of a novel class of PHA copolymers. Biomacromolecules.

[9] Noda I., Lindsey S.B., Carraway D., Chen G.-Q. (2010). Nodax^TM^ class PHA copolymers. Plastics from Bacteria: Natural Functions and Applications.

[10] Nguyen T.V., Nagata T., Noso K., Kaji K., Masunaga H., Hoshino T., Hikima T., Sakurai S., Yamamoto K., Miura Y. (2021). Effect of the 3-hydroxyhexanoate content on melt-isothermal crystallization behavior of microbial poly(3-hydroxybutyrate-co-3-hydroxyhexanate). Macromolecules.

[11] Adar F., Street R., Noda I. (2023). Isothermal crystallization of polyhydoxyalkanoate (PHA) utilizing Raman spectroscopy to follow chain packing as well as molecular motion. Spectrochim. Acta Part A Mol. Biomol. Spectrosc..

[12] Noda I. (1993). Generalized two-dimensional correlation method applicable to infrared, Raman, and other types of spectroscopy. Appl. Spectrosc..

[13] Noda I., Ozaki Y. (2004). Two Dimensional Correlation Spectroscopy: Applications in Vibrational and Optical Spectroscopy.

[14] Noda I., Dowrey A.E., Marcott C., Story G.M., Ozaki Y.J.A.S. (2000). Generalized two-dimensional correlation spectroscopy. Appl. Spectrosc..

[15] Sobieski B.J., Noda I., Rabolt J.F., Chase D.B. (2017). Observation of intermolecular hydrogen bonding interactions in biosynthesized and biodegradable poly[(R)-3-hydroxybutyrate-*co*-(R)-3-hydroxyhexanoate] in chloroform and 1,1,1,3,3,3-hexafluoro-2-propanol (HFIP). Appl. Spectrosc..

[16] Padermshoke A., Katsumoto Y., Sato H., Ekgasit S., Noda I., Ozaki Y. (2004). Surface melting and crystallization behavior of polyhydrxyalkanoates studied by attenuated total reflection infrared spectroscopy. Polymer.

[17] Padermshoke A., Sato H., Katsumoto Y., Ekgasit S., Noda I., Ozaki Y. (2004). Crystallization behavior of poly(3-hydrxybutyrate-*co*-3-hydroxyhexanoate) studied by 2D IR correlation spectroscopy. Polymer.

[18] Padermshoke A., Sato H., Katsumoto Y., Ekgasit S., Noda I., Ozaki Y. (2004). Thermally induced phase transition of poly(3-hydrxybutyrate-*co*-3-hydroxyhexanoate) investigated by two-dimensional correlation spectroscopy. Vib. Spectrosc..

[19] Padermshoke A., Katsumoto Y., Ekgasit S., Noda I., Ozaki Y. (2005). Melting behavior of poly(3-hydrxyalkanoate) investigated by two-dimensional infrared correlation spectroscopy. Spectrochim. Acta Part A.

[20] Noda I., Roy A., Carriere J., Sobieski B.J., Chase D.B., Rabolt J.F. (2017). Two-dimensional Raman correlation spectroscopy study of poly[(R)-3-hydroxybutyrate-*co*-(R)-3-hydroxyhexanoate] copolymer. Appl. Spectrosc..

[21] Sato H., Murakami R., Padermshoke A., Hirose F., Senda K., Noda I., Ozaki Y. (2004). Infrared spectroscopy study of CH⋯O hydrogen bondings and thermal behavior of biodegradable poly(hydroxyalkanoate). Macrolecules.

[22] Sato H., Mori K., Murakami R., Ando Y., Takahashi I., Zhang J., Terauchi H., Hirose F., Senda K., Tashiro K. (2006). Crystal and lamella structure and CH⋯O=C hydrogen bonding and thermal behavior of poly(3-hydroxyalkanoate) studied by x-ray diffraction and infrared spectroscopy. Macrolecules.

[23] Wang H., Tashoro K. (2016). Reinvestigation of crystal structure and intermolecular interactions of biodegradable poly(3-hydroxybutyrate) α-form and the prediction of its molecular property. Macromolecules.

[24] Brela M.Z., Boczar M., Wójcik M.J., Sat H., Nakajima T., Ozaki Y. (2017). The Born-Oppenheimer molecular simulations of infrared spectra of crystalline poly-(R)-3-hydroxybutyrate with analysis of weak C–H⋯O=C hydrogen bonds. Chem. Phys. Lett..

